# Resveratrol Improves Hepatic Redox Status and Lipid Balance of Neonates with Intrauterine Growth Retardation in a Piglet Model

**DOI:** 10.1155/2020/7402645

**Published:** 2020-07-18

**Authors:** Kang Cheng, Shuli Ji, Peilu Jia, Hao Zhang, Ting Wang, Zhihua Song, Lili Zhang, Tian Wang

**Affiliations:** College of Animal Science and Technology, Nanjing Agricultural University, Nanjing 210095, China

## Abstract

Abnormal lipid metabolism, oxidative stress (OS), and inflammation play a pivotal role in the increased susceptibility to neonatal fatty liver diseases associated with intrauterine growth retardation (IUGR). This study was firstly conducted to investigate whether resveratrol could alleviate IUGR-induced hepatic lipid accumulation, alteration of redox and immune status in a sucking piglet model and explore the possible mechanisms at transcriptional levels. A total of 36 pairs of 7 d old male normal birth weight (NBW) and IUGR piglets were orally fed with either 80 mg resveratrol/kg body weight/d or 0.5% carboxymethylcellulose sodium for a period of 14 days, respectively. Compared with the NBW piglets, the IUGR piglets displayed compromised growth performance and liver weight, reduced plasma free fatty acid (FFA) level, increased hepatic OS, abnormal hepatic lipid accumulation and weakened hepatic immune function, and hepatic aberrant transcriptional expression of some genes such as heme oxygenase 1, superoxide dismutase 1, sterol regulatory element-binding protein 1c, stearoyl-CoA desaturase 1, liver fatty acid-binding proteins 1, toll-like receptor 4, and tumor necrosis factor alpha (TNF-*α*). Oral administration of resveratrol to piglets decreased the levels of FFA and total triglycerides (TG) in the plasma and hepatic TNF-*α* concentration, and increased glutathione reductase activity and reduced glutathione level in the liver. Resveratrol restored the increased alanine aminotransferase activity in the plasma of IUGR piglets. Treatment with resveratrol ameliorated the increased hepatic malondialdehyde, protein carbonyl, TG, and FFA concentrations induced by IUGR. Resveratrol treatment alleviated the reduced lipoprotein lipase activity and its mRNA expression as well as TNF-*α* gene expression in the liver of IUGR piglets. Hepatic glutathione peroxidase 1 and monocyte chemotactic protein 1 genes expression of piglets was upregulated by oral resveratrol administration. In conclusion, resveratrol administration plays a beneficial role in hepatic redox status and lipid balance of the IUGR piglets.

## 1. Introduction

Metabolic syndrome (MS) is a cluster of metabolic disorders including obesity, hypertension, dyslipidemia, hyperglycemia, and insulin resistance [1, 2] and is a contributor to the deaths resulting from noncommunicable diseases such as diabetes and cardiovascular disease [2]. In the etiology of these diseases, excessive fat storage in nonadipose tissues (e.g., liver) is a risk factor [3]. Ectopic deposition of lipid in hepatocytes is the result of an increased free fatty acid input correlated with its inefficient *β*-oxidation, esterification, or both [4, 5]. Of note, excellent antioxidative and immune status is beneficial for the biological oxidative phosphorylation of glycolipid substrate, which is very important for maintaining lipid balance. With the exception of diet and lifestyle, birth weight (BW) is also regarded as the main link between abnormal accumulation of lipid in the liver and the increased incidence of MS and its related diseases [6]. A large number of epidemiological and animal studies have shown that lower BW induced by intrauterine growth retardation (IUGR) is associated with elevated risk for the development of nonalcoholic fatty liver disease (NAFLD) in both children and adults [7–10]. Pigs were widely used as an animal model for IUGR studies in humans due to their biological similarity to humans [11]. Furthermore, the spontaneous occurrence of IUGR in pigs is similar to that of humans, which is mainly caused by placental insufficiency [12, 13]. In previous studies, IUGR pigs exhibited insulin resistance [14, 15] and increased lipid level [15, 16], oxidative stress (OS) [16, 17], and abnormal inflammation [18, 19] in the liver from birth to adulthood at different stages of growth. Therefore, improving hepatic lipid metabolism, redox and immune status in an IUGR piglet model may help in developing new strategies for IUGR infants to prevent or/and slow the progression of NAFLD.

Resveratrol (RSV, 3, 5, 4′-trihydroxystilbene) was isolated from various type of plants such as peanuts, grapes, and *Polygonum cuspidatum*. A body of evidence in preclinical studies showed that resveratrol can treat high-fat diet-induced NAFLD by decreasing OS, inhibiting inflammation, and regulating lipid metabolism [4, 5]. Of course, RSV has been reported to exert a critical role in diet-induced MS associated with disease models such as cognitive impairment [20], osteoarthritis [21], nephropathy [22], and cardiovascular disease [23]. However, until now, no available research has been conducted to investigate the beneficial effect of RSV on the status of lipid, redox, and immunity in the liver of IUGR piglets. Therefore, the effect of RSV on hepatic lipid metabolism, OS, and inflammation of IUGR newborn piglets was firstly explored.

## 2. Materials and Methods

The experiment was carried out according to the guidelines of the Ethics Committee of Nanjing Agricultural University for the use of animals in research.

### 2.1. Animal and Experimental Design

During the preparation, healthy sows (Landrace × Yorkshire) with the same parity of the third and similar expected days of farrowing (<3 d) were chosen. At birth, sows that had similar litter sizes (i.e., 11–13 piglets) and met the selection criteria for IUGR piglets were chosen. A total of 72 male newborn piglets (Duroc × (Landrace × Yorkshire)) were collected and tagged from 36 litters (1 normal birth weight (NBW) piglet and 1 IUGR piglet from 1 litter) for the experiment: 36 were NBW piglets (~1.72 kg) and the other 36 were naturally occurring IUGR littermates (~0.88 kg) according to their birth weight using our previous method [24]. An IUGR piglet was defined as having a birth weight which was 2 SD below the mean BW of the total population, whereas a NBW littermate had a birth weight within 0.5 SD unit of the mean birth weight of the whole litter. The NBW and IUGR piglets were cross-fostered after birth by 24 four-parity sows (standardized litters: 3 experimental piglets and 8 same type nonexperimental piglets). At 7 days of age, the NBW and IUGR piglets were orally fed with 80 mg RSV/kg body weight/d (purity 98%; Zhejiang Yixin Pharmaceutical Co., Ltd, China; diluted in 0.5% carboxymethylcellulose sodium (CMC-Na)) or the same volume of 0.5% CMC-Na (Sinopharm Chemical Reagent Co., Ltd., Shanghai, China; diluted in 0.86% saline) for a period of 14 days, respectively. Therefore, all piglets were assigned into 4 groups (6 replicate per group, 3 piglets per replicate): NBW-CON, NBW-RSV, IUGR-CON, and IUGR-RSV. During the whole experimental period, all piglets remained with sows. The whole animal experiment was conducted in an experimental farm of Anyou Biotechnology Group (Taicang, China). During the pregnancy and lactation, sows are fed and managed according to standard procedures of the experimental farm. The piglets' average body weight (ABW) was recorded carefully.

### 2.2. Sample Collection

At 21 days of postnatal age, 1 piglet per replicate (6 piglets per treatment) was selected to collect a heparinized blood sample from the anterior vena cava after fasting for 8 h. The plasma was obtained by centrifugation at 2000×g for 20 min at 4°C and stored at -20°C until assays. After then, these piglets were killed as we previously described [25], and the liver (right lobe) sample was immediately collected and stored at -80°C for further analysis.

### 2.3. Plasma Biochemical Parameter Analysis

In the plasma, total triglycerides (TG), total cholesterol (TC), high-density lipoprotein cholesterol (HDL-C), low-density lipoprotein cholesterol (LDL-C), glucose (Glu) and free fatty acid (FFA) levels, and alanine aminotransferase (ALT) and aspartate aminotransferase (AST) activities were determined by commercial kits (Nanjing Jiancheng Institute of Bioengineering, Nanjing, China). A commercial enzyme-linked immunosorbent assay (ELISA) kit (CUSABIO Biotech, Wuhan, China) was used to measure the plasma insulin level. The sensitivity limit of insulin determination was 1 *μ*IU/mL, and the inter- and intra-assay coefficients of variation were less than 15%. The homeostasis model assessment of insulin resistance (HOMA-IR) was used to calculate insulin resistance according to the following formula: HOMA‐IR = fasting plasma insulin (*μ*U/mL) × fasting plasma glucose (mmol/L)/22.5 [26].

### 2.4. Hepatic Lipid Metabolism Parameters

The levels of TG, TC, and FFA and the activities of lipoprotein lipase (LPL), hepatic lipase (HL), and total lipase (TL) in the liver were determined according to the instructions of the manufacturer (Nanjing Jiancheng Institute of Bioengineering, Nanjing, China). These results were normalized to the total protein concentration in each sample for intersample comparison. Hepatic protein concentration was detected according to the Bradford method [27].

### 2.5. Determination of Hepatic Redox Status

The contents of malondialdehyde (MDA), protein carbonyl (PC), and reduced glutathione (GSH) and the activities of total superoxide dismutase (T-SOD), glutathione peroxidase (GPX), and glutathione reductase (GR) were measured by using assay kits purchased from the Nanjing Jiancheng Institute of Bioengineering (Nanjing, China) following the guidelines of the manufacturer. All parameters related to redox status were normalized against protein content in each sample for intersample comparison. The protein concentrations in the liver homogenate were quantified following the Bradford method [27].

### 2.6. Hepatic Cytokine Concentration Assays

The production of tumor necrosis factor alpha (TNF-*α*) was measured using an ELISA kit (Beijing Solarbio Science & Technology Co., Ltd., Beijing, China), which was performed according to the manufacturer's protocol. The detection limits were 5 pg/mL; the inter- and intra-assay coefficients of variation were less than 10%. All results were normalized to the total protein concentration in each sample for intersample comparison. Hepatic protein concentration was detected according to the Bradford method [27].

### 2.7. mRNA Expression

Total RNA isolation, reverse transcription, and quantitative real-time PCR (qRT-PCR) analysis were carried out as described previously [5, 28]. The primers used for qRT-PCR are presented in Table 1. The 2^−*ΔΔ*Ct^ method was used to calculate the mRNA expression levels of target genes relative to the housekeeping gene *β*-actin, as described previously [29]. The values of the NBW-CON group were regarded as a calibrator.

### 2.8. Statistical Analysis

Two-way ANOVA was used to determine the main effects (BW and RSV) and their interaction using the general linear model procedure of SPSS software (version 20.0; SPSS Inc.). When a significant interaction between BW and RSV treatment was observed, post hoc testing was conducted using Turkey's multiple comparison test. The ndividual piglet was used as the experimental unit with the exception of growth performance. Data are presented as means with their standard errors. Differences were considered significant at *P* < 0.05, and 0.05 < *P* < 0.10 were considered a trend.

## 3. Results

### 3.1. RSV Does Not Affect the Growth Performance but Improves the Circulatory Metabolism of Piglets

The IUGR piglets had lower ABW and liver weight but exhibited higher liver relative weight compared with the NBW piglets (*P* < 0.05, Table 2). However, RSV had no effects on the growth performance and organ weight of the piglets (*P* > 0.05). There is a significant interaction between BW and RSV treatment for the plasma TG level (*P* < 0.05, Table 3). Oral RSV reduced (*P* < 0.05) the level of plasma TG in the NBW piglets rather than in the IUGR piglets. Compared with NBW, IUGR decreased the concentration of FFA in the plasma of piglets (*P* < 0.05). Oral administration of RSV decreased the levels of FFA (*P* < 0.05), TG (*P* < 0.05), and Glu (*P* = 0.061) in the plasma of piglets. However, BW and RSV had no effects on HDL-C, LDL-C, TC, insulin, and HOMA-IR levels (*P* > 0.05).

### 3.2. RSV Alleviates Hepatic Injury of IUGR Piglets

**Figure 1:**
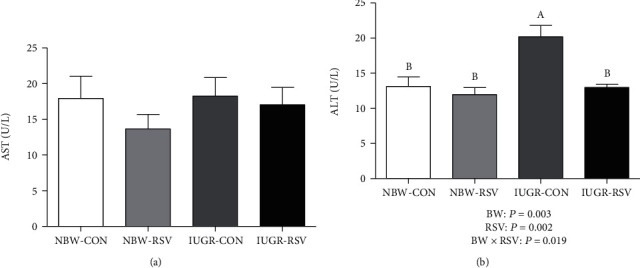
Aminotransferase activities in plasma of piglets at 21 days of age. (a) AST: aspartate aminotransferase; (b) ALT: alanine aminotransferase. BW: birth weight; RSV: resveratrol; NBW-CON: normal birth weight piglets orally fed with 0.5% carboxymethylcellulose sodium; NBW-RSV: normal birth weight piglets orally fed with 80 mg RSV/kg body weight/d; IUGR-CON: intrauterine growth-retarded piglets orally fed with 0.5% carboxymethylcellulose sodium; IUGR-RSV: intrauterine growth-retarded piglets orally fed with 80 mg RSV/kg body weight/d. The column and its bar represented the means value and standard error, *n* = 6, respectively. Means without a common letter differ significantly (*P* < 0.05).

Aminotransferase activities in the circulatory system are considered as reliable markers of hepatic damage. We measured ALT and AST activities in the plasma and found that BW and RSV treatment had a significant interaction effect on the plasma ALT activity in piglets (*P* < 0.05, Figure 1(b)). Resveratrol restored the increased ALT activity in the plasma of IUGR piglets (*P* < 0.05). There was no significant difference in the plasma AST activity of piglets among these groups (*P* > 0.05, Figure 1(a)).

### 3.3. RSV Affects the Hepatic Lipid Metabolism of Piglets

**Figure 2:**
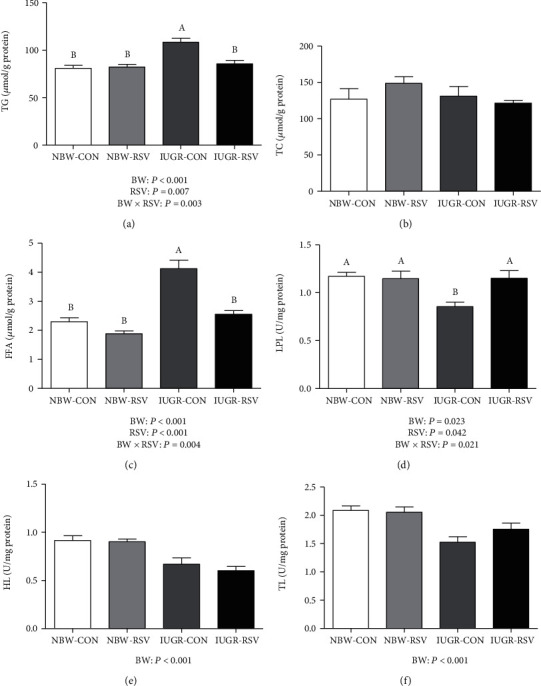
Hepatic lipid metabolism parameters of piglets at 21 days of age. (a) TG: total triglycerides; (b) TC: total cholesterol; (c) FFA: free fatty acids; (d) LPL: lipoprotein lipase; (e) HL: hepatic lipase; (f) TL: total lipase; BW: birth weight; RSV: resveratrol; NBW-CON: normal birth weight piglets orally fed with 0.5% carboxymethylcellulose sodium; NBW-RSV: normal birth weight piglets orally fed with 80 mg RSV/kg body weight/d; IUGR-CON: intrauterine growth-retarded piglets orally fed with 0.5% carboxymethylcellulose sodium; IUGR-RSV: intrauterine growth-retarded piglets orally fed with 80 mg RSV/kg body weight/d. The column and its bar represented the means value and standard error, *n* = 6, respectively. Means without a common letter differ significantly (*P* < 0.05).

The imbalance of lipid metabolism is an important driver to hepatic dysfunction and damage of the IUGR individuals. Thus, we evaluated the effect of RSV on the hepatic lipid metabolism of the IUGR piglets. Compared with the NBW piglets, IUGR increased TG and FFA levels and decreased LPL, HL, and TL activities in the liver of piglets (*P* < 0.05, Figures 2(a) and 2(c)–2(f)). Administration of RSV to piglets reduced TG and FFA levels and increased LPL activity in the liver (*P* < 0.05). In addition, BW and RSV treatment had significant interaction effects on LPL activity and the levels of TG and FFA in the liver of piglets (*P* < 0.05). Resveratrol treatment ameliorated the increased TG and FFA levels and the reduced LPL activity in the liver of IUGR piglets (*P* < 0.05).

**Figure 3:**
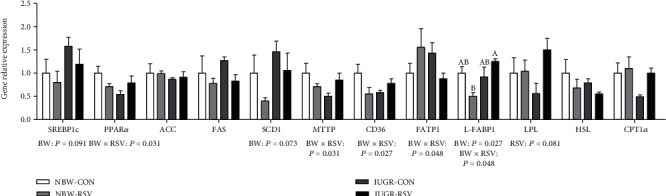
Genes expression related to lipid metabolism in the liver of piglets at 21 days of age. BW: birth weight; RSV: resveratrol; SREBP1c: sterol regulatory element-binding protein 1c; PPAR*α*: peroxisome proliferator-activated receptor alpha; ACC: acetyl-CoA carboxylase; FAS: fatty acid synthase; SCD1: stearoyl-CoA desaturase 1; MTTP: microsomal triglyceride transfer protein; CD36: cluster of differentiation 36; FATP1: fatty acid transport proteins 1; L-FABP1: liver fatty acid-binding proteins 1; LPL: lipoprotein lipase; HSL: hormone sensitive lipase; CPT1*α*: carnitine palmitoyltransferase 1 alpha; NBW-CON: normal birth weight piglets orally fed with 0.5% carboxymethylcellulose sodium; NBW-RSV: normal birth weight piglets orally fed with 80 mg RSV/kg body weight/d; IUGR-CON: intrauterine growth-retarded piglets orally fed with 0.5% carboxymethylcellulose sodium; IUGR-RSV: intrauterine growth-retarded piglets orally fed with 80 mg RSV/kg body weight/d. The column and its bar represented the means value and standard error, *n* = 5, respectively. Means without a common letter differ significantly (*P* < 0.05).

The mRNA abundance of sterol regulatory element-binding protein 1c (SREBP1c, *P* = 0.091, Figure 3), stearoyl-CoA desaturase 1 (SCD1, *P* = 0.073), and liver fatty acid-binding proteins 1 (L-FABP1, *P* < 0.05) were increased by IUGR compared with NBW. A tendency for the increased (*P* = 0.081) expression of the hepatic LPL gene was found in the piglets exposed to RSV treatment. A significant interaction (*P* < 0.05) between BW and RSV treatment was observed in the hepatic mRNA expression of peroxisome proliferator-activated receptor alpha, microsomal triglyceride transfer protein, cluster of differentiation 36, fatty acid transport proteins 1, and L-FABP1. No significant differences (*P* > 0.05) were observed in the hepatic genes expression of acetyl-CoA carboxylase (ACC), fatty acid synthase (FAS), hormone-sensitive lipase and carnitine palmitoyltransferase 1 alpha, and TC level (Figure 2(b)) among these groups.

### 3.4. RSV Improves the Hepatic Redox Status of Piglets

**Figure 4:**
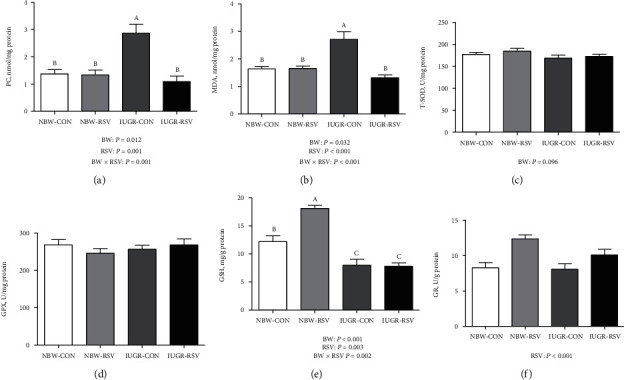
Redox status in the liver of piglets at 21 days of age. (a) PC: protein carbonyl; (b) MDA: malondialdehyde; (c) T-SOD: total superoxide dismutase; (d) GPX: glutathione peroxidase; (e) GSH: reduced glutathione; (f) GR: glutathione reductase; BW: birth weight; RSV: resveratrol; NBW-CON: normal birth weight piglets orally fed with 0.5% carboxymethylcellulose sodium; NBW-RSV: normal birth weight piglets orally fed with 80 mg RSV/kg body weight/d; IUGR-CON: intrauterine growth-retarded piglets orally fed with 0.5% carboxymethylcellulose sodium; IUGR-RSV: intrauterine growth-retarded piglets orally fed with 80 mg RSV/kg body weight/d. The column and its bar represented the means value and standard error, *n* = 6, respectively. Means without a common letter differ significantly (*P* < 0.05).

**Figure 5:**
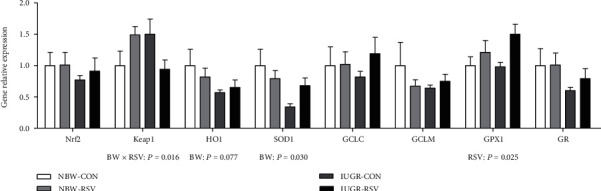
Genes expression related to antioxidation in the liver of piglets at 21 days of age. BW: birth weight; RSV: resveratrol; Nrf2: nuclear factor erythroid-derived 2-like 2; Keap1: Kelch-like ECH-associated protein 1; HO1: heme oxygenase 1; SOD1: superoxide dismutase 1; GCLC: glutamate-cysteine ligase catalytic subunit; GCLM: glutamate-cysteine ligase modifier subunit; GPX1: glutathione peroxidase 1; GR: glutathione reductase; NBW-CON: normal birth weight piglets orally fed with 0.5% carboxymethylcellulose sodium; NBW-RSV: normal birth weight piglets orally fed with 80 mg RSV/kg body weight/d; IUGR-CON: intrauterine growth-retarded piglets orally fed with 0.5% carboxymethylcellulose sodium; IUGR-RSV: intrauterine growth-retarded piglets orally fed with 80 mg RSV/kg body weight/d. The column and its bar represented the means value and standard error, *n* = 5, respectively. Means without a common letter differ significantly (*P* < 0.05).

Since OS is implicated in IUGR-induced liver injury, we also tested whether RSV improves hepatic redox status of the IUGR piglets. As shown in Figure 4, compared with the NBW piglets, the higher (*P* < 0.05) concentrations of MDA and PC, the lower (*P* = 0.096) T-SOD activity, and the reduced (*P* < 0.05) GSH level were observed in the IUGR piglets. Resveratrol treatment reduced MDA and PC contents and increased GR activity and GSH level in the liver of piglets (*P* < 0.05). There was an interaction between BW and RSV treatment for hepatic MDA, PC, and GSH levels (*P* < 0.05). Treatment with RSV ameliorated (*P* < 0.05) the increased hepatic MDA and PC concentrations induced by IUGR and increased (*P* < 0.05) hepatic GSH level in the NBW piglets rather than in the IUGR piglets. The mRNA expression of heme oxygenase 1 (HO1, *P* = 0.077, Figure 5) and SOD1 (*P* < 0.05) in the liver was downregulated by IUGR compared with NBW. Hepatic GPX1 gene expression was upregulated by oral RSV administration (*P* < 0.05). There was a significant interaction between BW and RSV treatment for the mRNA abundance of hepatic Kelch-like ECH-associated protein 1 (*P* < 0.05). There were no significant differences (*P* > 0.05) in hepatic genes expression of nuclear factor erythroid-derived 2-like 2, glutamate-cysteine ligase catalytic subunit, glutamate-cysteine ligase modifier subunit, and GR as well as GPX activity.

### 3.5. RSV Affects the Hepatic Immune Status of Piglets

**Figure 6:**
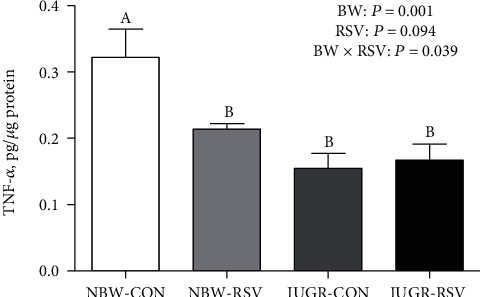
Tumor necrosis factor alpha (TNF-*α*) concentration in the liver of piglets at 21 days of age. BW: birth weight; RSV: resveratrol; NBW-CON: normal birth weight piglets orally fed with 0.5% carboxymethylcellulose sodium; NBW-RSV: normal birth weight piglets orally fed with 80 mg RSV/kg body weight/d; IUGR-CON: intrauterine growth-retarded piglets orally fed with 0.5% carboxymethylcellulose sodium; IUGR-RSV: intrauterine growth-retarded piglets orally fed with 80 mg RSV/kg body weight/d. The column and its bar represented the means value and standard error, *n* = 6, respectively. Means without a common letter differ significantly (*P* < 0.05).

**Figure 7:**
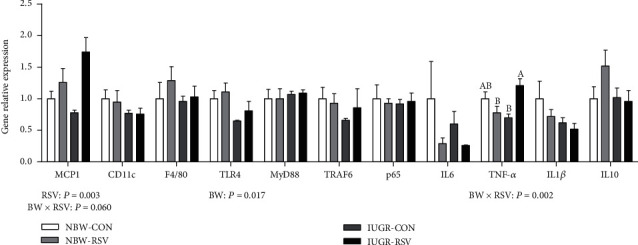
Genes expression related to inflammation in the liver of piglets at 21 days of age. BW: birth weight; RSV: resveratrol; MCP1: monocyte chemotactic protein 1; CD11c: integrin alpha X; F4/80: adhesion G protein-coupled receptor E1; TLR4: toll-like receptor 4; MyD88: myeloid differentiation factor 88; TRAF6: tumor necrosis factor receptor-associated factor 6; NF-*κ*B p65: nuclear factor-*κ*B; IL-6: interleukin 6; TNF-*α*: tumor necrosis factor alpha; IL1*β*: interleukin 1*β*; IL10: interleukin 10; NBW-CON: normal birth weight piglets orally fed with 0.5% carboxymethylcellulose sodium; NBW-RSV: normal birth weight piglets orally fed with 80 mg RSV/kg body weight/d; IUGR-CON: intrauterine growth-retarded piglets orally fed with 0.5% carboxymethylcellulose sodium; IUGR-RSV: intrauterine growth-retarded piglets orally fed with 80 mg RSV/kg body weight/d. The column and its bar represented the means value and standard error, *n* = 5, respectively. Means without a common letter differ significantly (*P* < 0.05).

Abnormal immune response is a key event in IUGR-induced liver injury. Therefore, we also studied the effect of RSV on hepatic inflammation of the IUGR piglets. Compared with the NBW piglets, IUGR decreased (*P* < 0.05, Figure 6) the hepatic TNF-*α* concentration in the piglets. Resveratrol reduced TNF-*α* concentration in the liver of piglets (*P* < 0.05). There was a significant interaction between BW and RSV treatment for hepatic TNF-*α* concentration (*P* < 0.05); RSV treatment to the NBW piglets led to a significant decrease in hepatic TNF-*α* concentration. Hepatic toll-like receptor 4 (TLR4) gene expression in the IUGR piglets was lower than those in the NBW piglets (*P* < 0.05, Figure 7). Gene expression of monocyte chemotactic protein 1 (MCP1) in the liver of piglets was increased by RSV administration (*P* < 0.05). There was an interaction between BW and RSV treatment for hepatic TNF-*α* (*P* < 0.05) and MCP1 (*P* = 0.060) genes expression. Resveratrol increased TNF-*α* gene expression in the liver of IUGR piglets (*P* < 0.05). Hepatic genes expression of CD11c, adhesion G protein-coupled receptor E1, myeloid differentiation factor 88, tumor necrosis factor receptor-associated factor 6, nuclear factor-*κ*B p65, interleukin 6 (IL6), IL1*β*, and IL10 were not affected (*P* > 0.05) among these groups.

## 4. Discussion

The occurrence of NAFLD in IUGR offspring endangers public health. NAFLD represents a spectrum of pathological changes from hepatic steatosis (fatty liver) without hepatocellular damage to nonalcoholic steatohepatitis which is the extreme form of the disease characterized by excessive fat accumulation (triglycerides) in hepatocytes [30, 31]. Besides, OS and inflammation play an important role in the development of NAFLD subjects associated with IUGR [32]. Yamada et al. [33] reported that IUGR newborns persistently exhibited an increase in hepatic lipid content and upregulated lipogenic indices, which suggests that fatty liver occurs early in IUGR offspring. As expected, in the present study, hepatic dysfunction and injury in the IUGR piglets were evidenced by the increased plasma ALT activity, hepatic OS, and lipid accumulation, which were alleviated by oral RSV administration.

The accumulation of lipid in the liver is due to the fact that fatty acid uptake and synthesis exceed hepatocyte oxidative capacity [32]. The present study found that IUGR increased TG and FFA concentrations and the mRNA expression of SREBP1c, SCD1, and L-FABP1 in the liver of piglets. SREBP1c, a nuclear transcription regulator, can regulate lipogenic genes expression including ACC, SCD1, and FAS. SCD1 is a microsomal enzyme which catalyzes the synthesis of monounsaturated long-chain fatty acids from saturated fatty acyl-CoAs [34]. A previous study reported that liver-specific SCD1 inhibition resulted in decreased rates of fatty acid synthesis and downregulated expression of key lipogenic genes (e.g., FAS and ACC), partly through reduced transcription of SREBP1c [35]. Thus, the genes expression levels of SREBP1c and SCD1 are critical for hepatic *de novo* lipogenesis. L-FABP1 may facilitate the transport of fatty acids across the cellular membrane, and fatty acids may be involved in metabolic, inflammatory, and oxidative responses [34]. Interestingly, we also found that IUGR decreased hepatic LPL, HL, and TL activities of piglets. LPL and HL, members of the family of triglyceride lipase, are to catalyze the hydrolysis of triacylglycerol in circulating lipoproteins such as chylomicron and very low-density lipoprotein, providing FFA and glycerol for tissue storage or utilization [36]. The results of this study indicated that IUGR increased hepatic *de novo* lipogenesis and direct fatty acid uptake of piglets, leading to hepatic lipid accumulation. Similar results were obtained in an IUGR piglet model which was conducted by He et al. [15]. In addition, the reduced circulatory FFA level in IUGR piglets may be attributed to the increased uptake of fatty acid in the liver. Expectedly, RSV decreased hepatic TG and FFA concentrations and increased hepatic LPL activity and its gene expression of the IUGR piglets. However, RSV had no effects on the genes expression of lipogenesis in the liver of IUGR piglets. Zhang et al. [24] found that IUGR impaired mitochondrial biogenesis and energy homeostasis in the liver of piglets, and these negative influences were restored by RSV. Mitochondria are important organelles which are essential for energy generation and are the primary site for fatty acid *β*-oxidation [37], indicating that normal mitochondrial function plays a vital role in hepatic lipid equilibrium. This study suggested that the lipid-lowing effect of RSV on the liver of IUGR piglets may be through the improvement of mitochondrial function rather than the inhibition of lipid synthesis and uptake.

The balance of redox status in the organism is achieved by reactive oxygen species (ROS) production and antioxidant defense capacity and is essential for maintaining the normal function of organs (e.g., liver) [38]. The excessive generation of ROS damages lipids, proteins, and DNA due to the impairment of the antioxidant system [5], indicating that OS occurs. In the present study, hepatic MDA and PC levels were increased in the IUGR piglets, which was consistent with a previous study [25]. MDA and PC contents reflect the degree of lipid peroxidation and protein oxidation, respectively, and are regarded as the index of OS [39]. Obviously, in the present study, hepatic OS in the IUGR piglets was observed and may be due to the reduced T-SOD activity and GSH level. The antioxidant defense system in cells, including enzymatic (e.g., SOD) and nonenzymatic (e.g., GSH) antioxidants, protects the organism against oxidative damage [40]. SOD catalyzes the reduction of the superoxide anion to hydrogen peroxide, which is further decomposed into water and oxygen by GPX or/and catalase [5]. GSH with its sulfhydryl group functions in the maintenance of sulfhydryl groups of other molecules (especially proteins), as a catalyst for disulfide exchange reactions, and in the detoxification of foreign compounds and free radicals [41]. Similar to previous studies [25, 39], IUGR harms antioxidant levels in offspring, which partly leads to OS. In addition, another reason for hepatic OS of the IUGR piglets is the overproduction of mitochondrial ROS. Mitochondrial fatty acid oxidation increases in response to excessive hepatic fat accumulation [42], and then, the generation of ROS was elevated in the liver. Interestingly, the elevated hepatic TG and FFA concentrations were found in the IUGR piglets. Thus, the enhancement of antioxidant defense is beneficial for lipid balance and redox status. In the present study, RSV administration reduced MDA and PC concentrations and increased GR activity and GSH level in the liver of piglets. Similar results observed in rodents' studies demonstrated that RSV enhances the activities of various antioxidant enzymes (e.g., SOD and GPX) and the induction of GSH by regulating various signaling pathways including Nrf2 and nuclear factor *κ*B [43]. Zhang et al. [44] reported that dietary RSV supplementation increased antioxidative capacity, GPX activity, and its mRNA level in the longissimus dorsi of finishing pigs. In the present study, RSV treatment only upregulated hepatic GPX1 mRNA expression of piglets but had no effects on other related genes expressions, which indicated that RSV may improve hepatic redox status through the posttranslational protein modifications rather than the mRNA level regulation. The deeper molecular mechanism needs to be further investigated. In the present study, we speculated that RSV alleviated IUGR-induced increased MDA and PC concentrations in the liver of suckling piglets by reducing ROS production via the enhancement of hepatic mitochondrial function [24] and the improvement of redox status.

Anti- and pro-inflammatory cytokine levels generated by immune cells are very important to resist the invasion of foreign antigens. TLR4 is a member of toll-like receptor and mediates the innate immune responses. However, defects at the level of expression of TLR4 could contribute to poor recruitment of antigen-presenting cells, and T and B cells at the site of inflammation, resulting in suboptimal adaptive immune responses which leads to the increased risk of infections with Gram-negative bacteria [45]. In the present study, IUGR piglets exhibited decreased hepatic TNF-*α* content, which may be related to the reduced TLR4 and TNF-*α* genes expression. The results suggested that IUGR can impair the hepatic immune function of piglets and increase the risk of illness. Similar results were shown in a piglet study in which IUGR markedly decreased mRNA abundance of TLR9 and toll-interacting protein in the ileum of piglets during the suckling period [46]. In the present study, although RSV upregulated hepatic MCP1 and TNF-*α* genes expression in the IUGR piglets, hepatic TNF-*α* protein content in the IUGR piglets was not altered by RSV. However, it is lacking the effect of RSV on the inflammation of pigs. The underlying mechanisms involved in the beneficial effect of RSV on hepatic inflammation of the IUGR piglets need to be investigated in the future.

## 5. Conclusions

In conclusion, oral RSV treatment reduced hepatic fat accumulation and OS in the IUGR piglets, which may suggest a potential nutritional strategy to prevent or/and slow the development of NAFLD in human infants with IUGR.

## Figures and Tables

**Table 1:** Sequences for real-time PCR primers.

Gene	GenBank ID	Sequence (5′⟶3′, forward primer/reverse primer)	Product length (bp)
Nrf2	XM_013984303.2	ATCCAGCGGATTGCTCGTAG	155
		TCAAATCCATGTCCTTGGCG	
Keap1	NM_001114671.1	TCTGCTTAGTCATGGTGACCT	158
		GGGGTTCCAGATGACAAGGG	
HO1	NM_001004027.1	TGATGGCGTCCTTGTACCAC	71
		GACCGGGTTCTCCTTGTTGT	
SOD1	NM_001190422.1	CATTCCATCATTGGCCGCAC	118
		TTACACCACAGGCCAAACGA	
GPX1	NM_214201.1	CCTCAAGTACGTCCGACCAG	85
		GTGAGCATTTGCGCCATTCA	
GCLC	XM_003482164.4	CTAGTGGGTAGGCGGACTGG	81
		CGGTGTCGTGCTCTAGCTTC	
GCLM	XM_001926378.4	GGACAAAACCCAGTTGGAGC	86
		TCACACAGCAAGAGGCAAGA	
GR	AY368271.1	GTGAGCCGACTGAACACCAT	141
		CAGGATGTGAGGAGCTGTGT	
SREBP1c	NM_214157.1	GCGACGGTGCCTCTGGTAGT	218
		CGCAAGACGGCGGATTTA	
PPAR*α*	NM_001044526.1	GGCACTGAACATCGAATGTAGAAT	80
		TGCAACCTTCACAGGCATGA	
ACC	NM_001114269.1	ATCCCTCCTTGCCTCTCCTA	208
		ACTTCCCGTTCAGATTTCCG	
FAS	NM_001099930.1	TACCTTGTGGATCACTGCATAGA	113
		GGCGTCTCCTCCAAGTTCTG	
SCD1	NM_213781.1	ATTGGGAGCTGTGGGTGAG	90
		AAGTTGATGTGCCAGCGGTA	
HSL	NM_214315.3	GCAGCATCTTCTTCCGCACA	195
		AGCCCTTGCGTAGAGTGACA	
CPT1*α*	NM_001129805.1	TCAAAAACGGCAAGATGGGC	155
		TGGAATGTTGGGGTTGGTGT	
LPL	NM_214286.1	CACATTCACCAGAGGGTC	177
		TCATGGGAGCACTTCACG	
L-FABP1	NM_001004046.2	AGGGGACATCGGAAATCGTG	103
		TCACACTCCTCTCCCAAGGT	
CD36	NM_001044622.1	TGACCCAGCACTTGAAGCAA	130
		AAGATATCAGTTAGGAGTCCGATGA	
FATP1	NM_001083931.1	AGGTCTGGCGTGGGTCAAAG	208
		GGAGTAGAGGGCAAAGCAGG	
MTTP	NM_214185.1	AGCAAAATGGTCCGTCGAGT	114
		CGAATGGGGACCACGTTCTA	
MCP1	NM_214214.1	AAACGGAGACTTGGGCACAT	74
		GCAAGGACCCTTCCGTCATC	
F4/80	XM_021083974.1	TCCTTCTCTTTTGGGGGTGT	73
		GCCATTGACTCCAACGGAGA	
CD11c	XR_002342355.1	GGAGCAAATGGACAGACCGT	95
		GAATGCAGGTGCAAAGGCAA	
TLR4	GQ304754	TTTCTTGCAGTGGGTCGAGG	161
		GGAAGGTGAGAACTGACGCA	
MyD88	NM001099923.1	GTGCCGTCGGATGGTAGTG	65
		TCTGGAAGTCACATTCCTTGCTT	
TRAF6	NM_001105286.1	GCTGCATCTATGGCATTTGAAG	71
		CCACAGATAACATTTGCCAAAGG	
NF-KB, p65	NM_001114281.1	GGGGCGATGAGATCTTCCTG	110
		CACGTCGGCTTGTGAAAAGG	
TNF-*α*	NM_214022.1	GCCCTTCCACCAACGTTTTC	97
		CAAGGGCTCTTGATGGCAGA	
IL6	NM_214399.1	ACAAAGCCACCACCCCTAAC	185
		CGTGGACGGCATCAATCTCA	
IL1*β*	NM_214055.1	ATTCAGGGACCCTACCCTCTC	92
		ATCACTTCCTTGGCGGGTTC	
IL10	NM_214041.1	CGGCCCAGTGAAGAGTTTCT	98
		GGCAACCCAGGTAACCCTTA	
*β*-Actin	XM_003124280.5	CTCCAGAGCGCAAGTACTCC	153
		AATGCAACTAACAGTCCGCC	

Nrf2: nuclear factor erythroid-derived 2-like 2; Keap1: Kelch-like ECH-associated protein 1; HO1: heme oxygenase 1; SOD1: superoxide dismutase 1; GPX1: glutathione peroxidase 1; GCLC: glutamate-cysteine ligase catalytic subunit; GCLM: glutamate-cysteine ligase modifier subunit; GR: glutathione reductase; SREBP1c: sterol regulatory element-binding protein 1c; FAS: fatty acid synthase; ACC: acetyl-CoA carboxylase; SCD1: stearoyl-CoA desaturase 1; PPAR*α*, peroxisome proliferator-activated receptor alpha; HSL: hormone-sensitive lipase; CPT1*α*: carnitine palmitoyltransferase 1 alpha; LPL: lipoprotein lipase; L-FABP1: liver fatty acid-binding proteins 1; CD36: cluster of differentiation 36; FATP1: fatty acid transport proteins 1; MTTP: microsomal triglyceride transfer protein; MCP1: monocyte chemotactic protein 1; F4/80: adhesion G protein-coupled receptor E1; CD11c: integrin alpha X; TLR4: toll-like receptor 4; MyD88: myeloid differentiation factor 88; TRAF6: tumor necrosis factor receptor-associated factor 6; NF-*κ*B: nuclear factor-*κ*B; TNF-*α*: tumor necrosis factor alpha; IL-6: interleukin 6; IL1*β*: interleukin 1*β*; IL10: interleukin 10; *β*-actin: beta actin.

**Table 2:** Growth performance and organ weight of piglets at 21 days of age.

Items	NBW-CON	NBW-RSV	IUGR-CON	IUGR-RSV	*P* value		
					BW	RSV	BW × RSV
ABW (kg)	7.17 ± 0.18	6.94 ± 0.12	4.32 ± 0.09	4.45 ± 0.12	<0.001	0.995	0.337
Liver (g)	185.62 ± 7.22	190.52 ± 3.56	142.63 ± 12.81	164.15 ± 5.20	<0.001	0.114	0.311
Liver relative weight (g/kg)	25.43 ± 1.13	22.00 ± 0.39	26.78 ± 2.10	26.60 ± 0.89	0.032	0.176	0.219

ABW: average body weight; BW: birth weight; RSV: resveratrol; NBW-CON: normal birth weight piglets orally fed with 0.5% carboxymethylcellulose sodium; NBW-RSV: normal birth weight piglets orally fed with 80 mg RSV/kg body weight/d; IUGR-CON: intrauterine growth-retarded piglets orally fed with 0.5% carboxymethylcellulose sodium; IUGR-RSV: intrauterine growth-retarded piglets orally fed with 80 mg RSV/kg body weight/d. Results present as means and standard errors, *n* = 6.

**Table 3:** Glycolipid metabolism parameters in plasma of piglets at 21 days of age.

Items	NBW-CON	NBW-RSV	IUGR-CON	IUGR-RSV	*P* value		
					BW	RSV	BW × RSV
HDL-C (mmol/L)	1.97 ± 0.15	1.84 ± 0.12	1.70 ± 0.08	1.69 ± 0.23	0.192	0.662	0.715
LDL-C (mmol/L)	1.38 ± 0.24	0.88 ± 0.07	0.96 ± 0.29	0.85 ± 0.08	0.269	0.137	0.330
TG (mmol/L)	1.25 ± 0.19^a^	0.66 ± 0.05^b^	0.94 ± 0.12^ab^	0.95 ± 0.07^ab^	0.901	0.028	0.021
TC (mmol/L)	7.06 ± 0.96	5.47 ± 0.39	4.90 ± 0.71	5.38 ± 0.45	0.107	0.416	0.136
FFA (*μ*mol/L)	200 ± 24.70	116.71 ± 12.65	135.44 ± 4.70	81.73 ± 2.80	0.002	<0.001	0.308
Glu (mmol/L)	20.49 ± 1.76	18.99 ± 1.45	21.83 ± 1.73	16.47 ± 1.93	0.736	0.061	0.278
Insulin (*μ*IU/mL)	31.89 ± 13.20	23.83 ± 5.79	26.40 ± 9.02	24.43 ± 6.34	0.791	0.586	0.741
HOMA-IR	28.27 ± 10.80	20.11 ± 5.58	26.00 ± 9.37	18.15 ± 4.75	0.795	0.331	0.985

FFA: free fatty acids; Glu: glucose; HDL-C: high-density lipoprotein cholesterol; HOMA-IR: homeostasis model assessment of insulin resistance; LDL-C: low-density lipoprotein cholesterol; TC: total cholesterol; TG: total triglycerides; BW: birth weight; RSV: resveratrol; NBW-CON: normal birth weight piglets orally fed with 0.5% carboxymethylcellulose sodium; NBW-RSV: normal birth weight piglets orally fed with 80 mg RSV/kg body weight/d; IUGR-CON: intrauterine growth-retarded piglets orally fed with 0.5% carboxymethylcellulose sodium; IUGR-RSV: intrauterine growth-retarded piglets orally fed with 80 mg RSV/kg body weight/d. Results present as means and standard errors, *n* = 6. Means in a row without a common letter differ significantly (*P* < 0.05).

## Data Availability

The data used to support the findings of this study are available from the corresponding authors upon request.
